# Validation of blood flow partitioning in 4D phase contrast CMR measurements using lagrangian coherent structures

**DOI:** 10.1186/1532-429X-13-S1-O24

**Published:** 2011-02-02

**Authors:** Johannes Töger, Marcus Carlsson, Gustaf Söderlind, Håkan Arheden, Einar Heiberg

**Affiliations:** 1 Skåne University Hospital, Lund University, Lund, Sweden; 2 Department of Numerical Analysis, Lund University, Lund, Sweden

## Introduction

Intracardiac blood flow measurements using 4D Phase Contrast CMR (4D PC-CMR) may contain useful information about cardiac pumping. Lagrangian Coherent Structures (LCS) is a new, operator-independent method which can simplify analysis by partitioning the flow into regions with different origins and destinations. Since LCS are operator-independent, they may be used to define quantitative indices of intracardiac blood flow. The partitioning has not previously been validated against particle tracing in 4D PC-CMR blood flow.

## Purpose

To investigate whether Lagrangian Coherent Structures computed from 4D Phase Contrast CMR of the human heart partitions diastolic inflow blood from blood already in the left ventricle.

## Methods

Eight healthy volunteers (5 male, 3 female, ages 23-63) underwent 4D PC-CMR flow measurements of the whole heart. Three-dimensional LCS surfaces were computed and automatically delineated in the left ventricle during diastole. The only parameter adjustable by the operator was the start of diastole. Particle tracing was performed to study the blood flowing into the ventricle during diastole and the blood already present in the ventricle from the previous heartbeat. LCS and particle traces were compared visually using the software Ensight (CEI, USA).

## Results

In all eight subjects, a distinct LCS was found in the left ventricle during diastole. The LCS separated the inflow blood particle traces and particle traces of blood already in the ventricle in all eight subjects. The Figure shows the typical appearance of the LCS and particle traces in one healthy volunteer.

**Figure 1:**
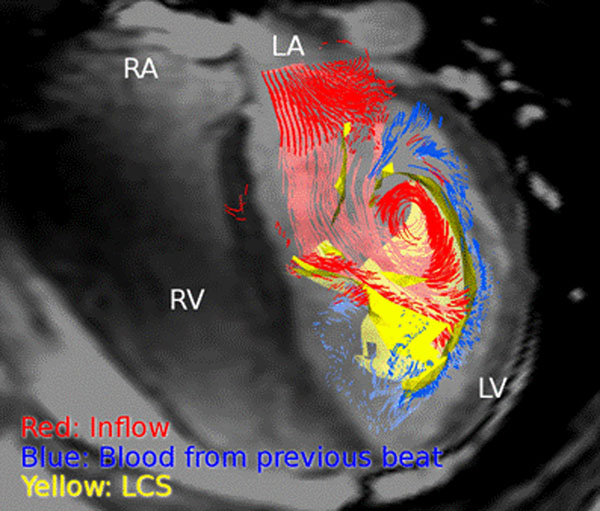
Lagrangian Coherent Structure (LCS, yellow) in the left ventricle of a healthy volunteer during mid-diastole. A transparent four-chamber image of the heart is shown for orientation. Red particle traces show blood flowing into the ventricle during diastole, and blue particles show blood already in the ventricle from the previous heartbeat. Note that the yellow LCS separates the blue and red particle traces. LV: Left Ventricle, RV: Right Ventricle, LA: Left Atrium, RA: Right Atrium.

## Conclusions

Lagrangian Coherent Structures can separate 4D Phase Contrast CMR data into regions of blood with different origins. Since Lagrangian Coherent Structures are operator-independent, they have the potential to be used to define quantitative indices of intracardiac blood flow.

